# Experimental Study on Uniaxial Compression Stress-Strain Relationship of Hybrid Fiber-Reinforced Coral Sand Ultra-High Performance Concrete

**DOI:** 10.3390/ma18102233

**Published:** 2025-05-12

**Authors:** Xiao Xue, Wei Li, Dongxu Hou, Hongwei Han, Yudong Han

**Affiliations:** 1 School of Water Conservancy and Civil Engineering, Northeast Agricultural University, Harbin 150030, China; 17691007992@163.com (X.X.); 18346238209@163.com (D.H.); 2 Central Research Institute of Building and Construction Co., Ltd., MCC Group, Beijing 100088, China; mcc_liwei@163.com.com; 3 Heilongjiang Provincial Key Laboratory of Water Resources and Water Conservancy Engineering in Cold Region, Northeast Agricultural University, Harbin 150030, China

**Keywords:** coral sand ultra-high-performance concrete, hybrid fiber, compressive constitutive model, radar chart analysis

## Abstract

The utilization of coral aggregates in the preparation of Ultra-High Performance Concrete (UHPC) effectively addresses the material scarcity challenges in island and reef construction environments, thereby advancing the sustainable development of building materials technology. This research systematically investigates the physical and mechanical properties of Coral Sand UHPC (CSUHPC) with varying fiber contents through uniaxial compression tests, splitting tensile tests, and stress–strain curve tests under compression. The experimental results demonstrate that the incorporation of fibers significantly enhances both the mechanical strength and ductility of CSUHPC. The test data indicate that CSUHPC specimens with a steel fiber volume fraction of 3% exhibit the highest performance, attaining a compressive strength of 131.9 MPa and a splitting tensile strength of 18.5 MPa. The compressive stress–strain curve tests reveal that the incorporation of fibers induces a failure mode transition in CSUHPC specimens from brittle to ductile. Furthermore, a constitutive equation for CSUHPC was proposed, and a multi-dimensional assessment system based on the radar chart, which encompasses compressive strength, splitting tensile strength, peak strain, compressive toughness, and an energy dissipation coefficient. The optimal fiber combination was determined as a hybrid fiber system comprising 2% steel fibers and 1% polyethylene (PE) fibers, which demonstrates superior comprehensive performance.

## 1. Introduction

The rapid development of tropical island regions is driving a growing demand for construction materials. Transporting sand and gravel from the mainland to these remote areas leads to significantly higher costs and extended project timelines [1,2]. When corals die, they are physically broken down into fragments primarily composed of calcium carbonate through natural hydrodynamic processes. Utilizing these coral fragments as aggregates in concrete production-replacing conventional sand and gravel can enhance resource efficiency and advance environmentally sustainable development strategies [3,4]. Research findings since the 20th century have demonstrated that coral concrete can satisfy the structural requirements of conventional buildings; however, its practical application has been limited by inherent material deficiencies such as cracking susceptibility, brittle fracture behavior, and compromised durability under mechanical loading [5,6,7]. Crack formation in coral concrete provides pathways for aggressive ions (e.g., chloride) to penetrate the matrix, accelerating internal corrosion and ultimately causing structural degradation. Furthermore, coral aggregates exhibit intrinsic limitations including elevated water absorption, high porosity, and reduced crushing strength—characteristics that critically hinder the widespread adoption of coral-based concrete in construction applications [8,9,10,11]. Tropical island environments are persistently characterized by extreme conditions—elevated temperatures, high humidity, saline saturation, and intense solar radiation. These synergistic stressors accelerate the degradation of concrete structures, manifesting as reduced compressive strength and compromised durability. Consequently, concrete materials in such regions demand enhanced mechanical performance and long-term resilience to withstand these multi-factor deterioration mechanisms [12,13,14,15]. UHPC demonstrates superior mechanical properties, enhanced durability, and improved workability compared to conventional concrete. These characteristics–including exceptional compressive strength, fracture resistance, and long-term stability–make UHPC particularly promising for marine engineering applications where structural resilience against corrosive environments is critical [16,17]. However, UHPC imposes stringent requirements for raw material quality and precise mix design during production, which consequently limits its applicability in engineering projects [18,19,20]. Consequently, this study investigates coral sand as an UHPC aggregate to achieve cost-effective marine infrastructure development.

Adding the right amount of fibers to concrete can reduce brittleness and increase strength [21]. At present, numerous scholars in the construction industry have achieved extensive research results in the study of single-mixed fiber concrete and its application to coral concrete [22,23,24]. Fu et al. incorporated basalt fibers into coral concrete, investigated the dynamic strength of the concrete under different confining pressures, and developed a dynamic constitutive model [25]. Liu studied the strength of cubic and prismatic carbon fiber-reinforced coral concrete specimens under uniaxial compression conditions [26]. Dai studied the effects of polypropylene, glass, and basalt fibers on the compressive strength of coral concrete, concluding that polypropylene fibers provided the most effective concrete enhancement [27]. Yue developed coral concrete with compressive strengths exceeding 70 MPa, and found that incorporating sisal fibers reduced concrete brittleness while exhibiting limited compressive strength enhancement [28]. Wang demonstrated that steel fiber reinforcement improved both embrittlement resistance and dynamic mechanical properties in coral concrete [29]. In marine–island environments, steel fibers exhibit susceptibility to chloride ion erosion, whereas organic fibers demonstrate vulnerability to UV-induced degradation under high radiation conditions. Consequently, researchers have utilized hybrid fiber systems in coral concrete formulations to investigate their synergistic effects on mechanical performance enhancement.

The above studies indicate that the incorporation of fibers can enhance the strength of concrete, but different fibers have distinct reinforcement mechanisms. Existing research has demonstrated that mixing multiple fibers in concrete can further enhance mechanical properties by leveraging the complementary characteristics of different fibers types [30,31,32,33]. For instance, Zhang studied the damage patterns of basalt-polypropylene fiber-reinforced coral concrete under impact loading, and concluded that hybrid fiber admixtures significantly affect the critical strain rate. Wang investigated steel-polyvinyl alcohol hybrid fibers in coral concrete and found a 46% increase in fracture energy compared to single-fiber groups at equivalent contents [34]. Liu explored carbon-basalt-steel fiber combinations, demonstrating further improvements in mechanical performance [35]. Current research efforts on coral concrete reveal limitations in achieving high mechanical strength, with hybrid fiber systems having been underexplored in coral concrete. This study proposes an innovative formulation of UHPC utilizing coral aggregates synergistically reinforced with steel fibers and PE fibers, thereby establishing a theoretical framework for enhancing both strength and toughness in marine–island infrastructure development.

In view of the above analysis, this study formulated a novel UHPC using coral sand as a fine aggregate. The variations in compressive strength, splitting tensile strength, and compressive stress-strain curves of CSUHPC with steel fibers (individually incorporated) and PE fibers (in hybrid combinations) were systematically investigated. The optimal fiber content was determined through the establishment of a multi-criteria evaluation system incorporating compressive strength, splitting tensile strength, peak strain, compressive toughness, and energy dissipation coefficient.

## 2. Materials and Methods

### 2.1. Raw Materials and Mix Proportions

**Figure 1:**
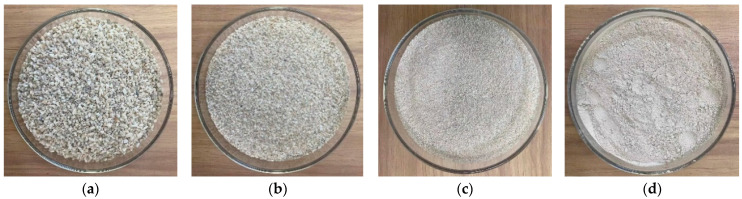
Coral sand of different particle sizes: (**a**) 1.25–2.5 mm; (**b**) 0.63–1.25 mm; (**c**) 0.315–0.63 mm; (**d**) 0–0.315 mm.

The raw materials used for preparing CSUHPC in this study included P-II 52.5 normal silicate cement, silica fume, and glass microspheres from Nanjing; coral sand collected and sieved from an island in the South China Sea, with a maximum particle size of 2.5 mm and a fineness modulus of 2.4. The main chemical composition of the coral sand is shown in Table 1, and its appearance is shown in Figure 1; Additionally, high-strength, high-modulus polyethylene fibers (PE fibers for short) produced in Beijing, and copper-plated steel fibers (Steel fibers for short) produced in Henan, were used. Their performance parameters are shown in Table 2. The study also employed a water reducer agent and defoamer, both produced in Tianjin. The dosage of the water reducer agent was 1.5% of the cementitious material, and the dosage of the defoamer was 10% of the water reducer agent dosage. The complete mix proportions are detailed in Table 3. Based on preliminary trials, fiber volume fractions exceeding 3% were found to significantly decrease both workability and strength. Therefore, the maximum fiber content was limited to 3% in this study.

### 2.2. Preparation of CSUHPC

The CSUHPC preparation process is divided into eight steps. Step 1: Coral sand, silica fume, glass microspheres, cement, and defoamer were dry-stirred in a mixer for 3 min until the powder was uniformly mixed. Step 2: The water reducer agent was added to the mixing water and thoroughly blended. The mixture was then poured into the mixer in two batches during slow stirring, each lasting 3 min. Step 3: Polyethylene (PE) fibers were pre-dispersed to prevent agglomeration, then gradually introduced into the mixture along with steel fibers under continuous mixing. Step 4: The mixture was further mixed for 3 minutes to ensure uniform fiber distribution. Step 5: Molds were coated with a release agent and the fresh mixture was poured into the prepared molds. Step 6: The filled molds were vibrated on a shaking table until no air bubbles emerged from the surface. Step 7: The molds were sealed with plastic film for 24 hours, followed by demolding. Step 8: Demolded specimens were cured in a standard chamber (20 °C ± 2 °C, RH ≥ 95%) until the designated age for subsequent testing.

**Figure 2:**
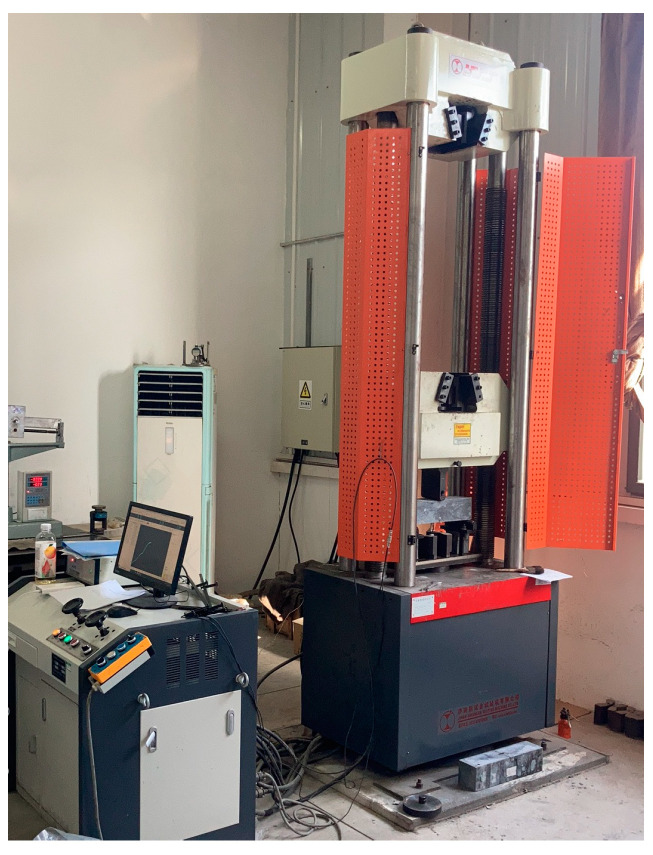
PC-controlled electro-hydraulic servo universal testing system WAW-1500.

All tests in this study were conducted using a WAW-1500 PC-controlled electro-hydraulic servo universal testing system (maximum load: 1500 kN) from the Central Research Institute of Building and Construction Co., Ltd., MCC Group (Beijing, China). The testing setup is illustrated in Figure 2.

### 2.3. Compressive Strength and Splitting Tensile Strength Tests of CSUHPC Cubes

**Figure 3:**
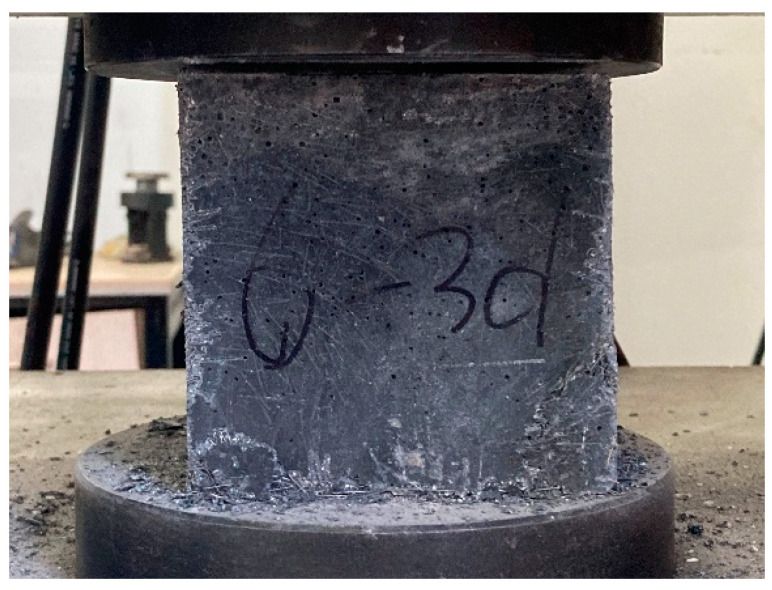
Compression strength test of CSUHPC cube.

**Figure 4:**
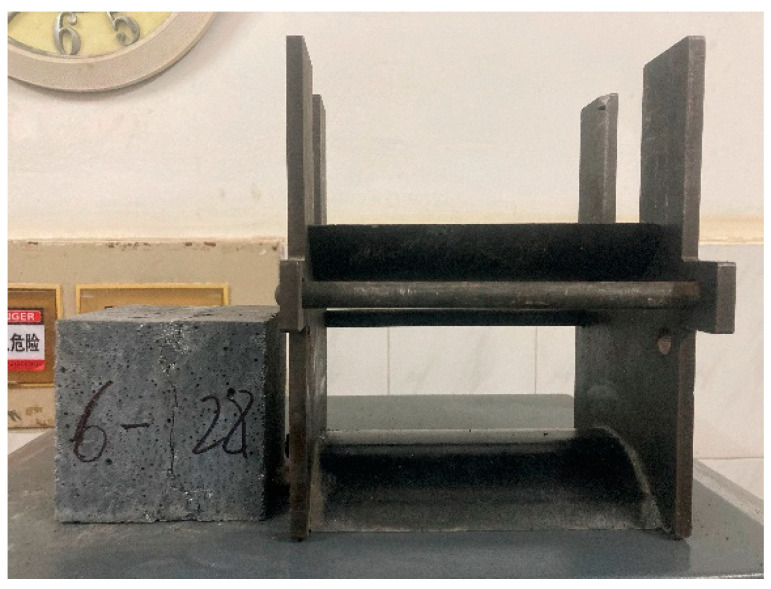
Splitting tensile strength test of CSUHPC cube.

In this study, strength tests were conducted on 100 mm cubic specimens [36,37]. The loading rates were 10 kN/s for compressive tests and 1 kN/s for splitting tensile tests. The compression strength test of the CSUHPC cube is shown in Figure 3, while the splitting tensile strength test is shown in Figure 4.

Concrete toughness can be measured by the tension–compression ratio, and concrete toughness is positively correlated with the tension–compression ratio. In this paper, the ratio of splitting tensile strength to compressive strength is defined as the tension–compression ratio. Additionally, to assess the role of fiber diameter, length, and admixture on the strength of CSUHPC specimens, the fiber reinforcement index (RI) is introduced [38]. The RI is calculated as the product of the fiber volume fraction and the fiber length-to-diameter ratio.

### 2.4. Compressive Stress–Strain Curve Test of CSUHPC Cylinders

**Figure 5:**
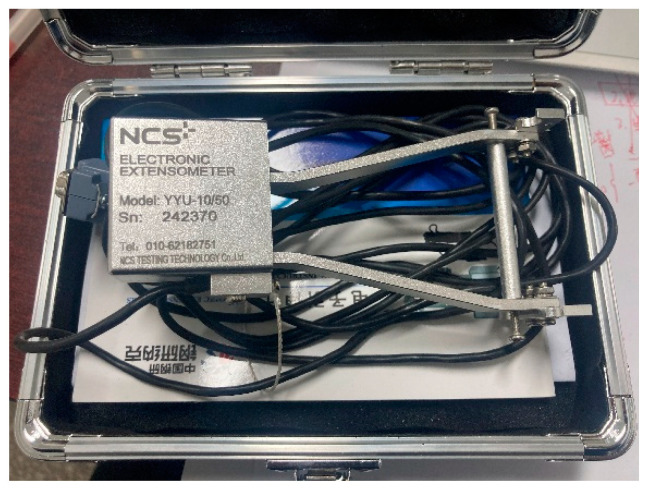
Electronic extensometer.

**Figure 6:**
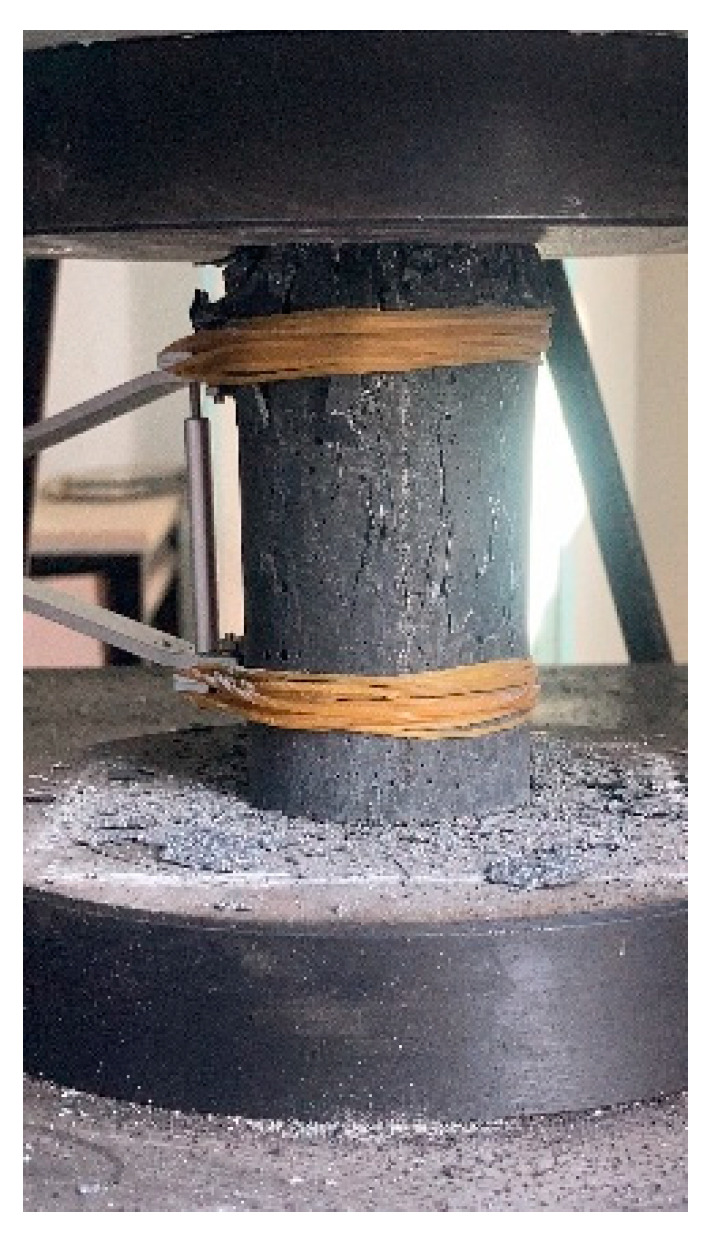
Cylindrical specimen compressive stress–strain curve testing.

The full compressive stress–strain curve was tested with reference to literature [39,40]. Cylinders of Φ50 mm × 100 mm were prepared. The specimen deformation data were collected using an NCS YYU-10/50 electronic extensometer from the NCS Testing Technology Co., Ltd. (Beijing, China), with a hydraulic press loading rate of 0.1 mm/min. The electronic extensometer is shown in Figure 5 and the compressive stress–strain curve test is shown in Figure 6. Since the extensometer is susceptible to specimen dislodgement during the strain data acquisition process, the strain data were corrected using the displacement data from the hydraulic press.

In order to measure the toughness of CSUHPC specimens, compressive toughness and energy dissipation coefficients were adopted [41,42]. Compressive toughness was calculated as the area under the compressive stress–strain curve, with the area under the curve up to the peak load. The energy dissipation coefficient was calculated as the ratio of the area enclosed by the stress–strain curve from the origin point to the descending section at 0.85 times the peak compressive strength and the coordinate axis, to the area of the corresponding strain rectangle from the origin point to the descending section at 0.85 times the peak compressive strength.

Concrete compressive stress–strain models can be categorized as unified and segmented. In this study, the stress and strain are first normalized, i.e., stress/peak stress ($y=\sigma_{c}/\sigma_{cp}$) and strain/peak strain ($x=\varepsilon_{c}/\varepsilon_{cp}$), and then different constitutive models were applied. The intrinsic equation for each stress model is shown below:

Carreira and Chu Unified Model (Model-1) [43]:

(1) $$y=\frac{nx}{n-1+n^{x}}$$

Zhenhai Guo Model (Model-2) [44]:

(2) $$y=\left\{\begin{array}{cc} ax+(3-2a)x^{2}+(a-2)x^{3} & 0\leq x\leq 1\\ \frac{x}{b{\left(x-1\right)}^{2}+x} & 1<x \end{array}\right.$$

Chuanbo Zhou Model (Model-3) [45]:

(3) $$y=\left\{\begin{array}{cc} ax+(5-4a)x^{4}+(3a-4)x^{5} & 0\leq x\leq 1\\ \frac{x}{b{\left(x-1\right)}^{2}+x} & 1<x \end{array}\right.$$

Deju Zhu Model (Model-4) [45]

(4) $$y=\left\{\begin{array}{cc} ax+(5-4a)x^{4}+(3a-4)x^{5} & 0\leq x\leq 1\\ \frac{x}{b{\left(x-1\right)}^{1.4}+x} & 1<x \end{array}\right.$$

The test compressive stress–strain curve is denoted as Exp. In the above equation, *n*, *a*, and *b* are parameters.

### 2.5. Performance Assessment

To systematically evaluate the comprehensive performance of CSUHPC specimens, this study adopts the radar assessment method to establish a multivariate evaluation system that includes cubic compressive strength, cubic splitting tensile strength, peak strain, compressive toughness, and an energy dissipation coefficient. Due to significant performance variations in the CSUHPC under different fiber volume fractions, all experimental data are normalized against their respective series’ peak values. These normalized datasets are subsequently used to construct radar charts for multi-parametric performance evaluation.

The radar map area *S_i_* and perimeter *C_i_* can be used as radar map features, and the vector (*A_i_,B_i_*) is constructed. Here, the *A_i_* value indicates the level of excellence in the mechanical properties of the specimen, while the *B_i_* value indicates the level of equilibrium in its mechanical properties. The geometric mean *F_i_* of the evaluation vector is introduced, where the *F_i_* value indicates the degree of excellence in the overall performance of the specimen. Each value is calculated using the following formula:

(5) $$S_{i}=\sum_{j=1}^{m}\frac{n_{ij}n_{i(j+1)}\sin\theta }{2}$$

(6) $$C_{i}=\sum_{j=1}^{m}\sqrt{n_{ij}^{2}+n_{i(j+1)}^{2}-2n_{ij}n_{i(j+1)}\cos\theta }$$

(7) $$A_{i}=\frac{S_{i}}{\max\left\{S_{i}\right\}}$$

(8) $$B_{i}=\frac{4\pi S_{i}}{C_{i}^{2}}$$

(9) $$F_{i}=\sqrt{A_{i}B_{i}}$$

where *m* is the number of assessment indicators, *n_ij_* is the *i*-th value in the *j*-th assessment object, and *θ* is the angle between adjacent ray axes of the radar map.

## 3. Results and Discussion

### 3.1. Strength Tests of CSUHPC Cubes

#### 3.1.1. Strength of CSUHPC Cubes

**Figure 7:**
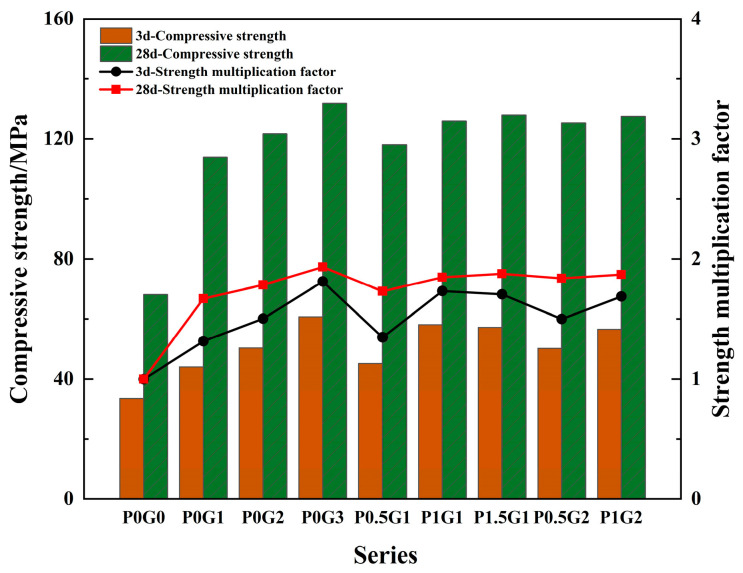
Compressive strength.

The compressive strength of CSUHPC with different fiber contents and curing ages is shown in Figure 7, where the folded line represents the growth rate. From this, the strength of CSUHPC increased with both fiber content and curing age. The cubic compressive strength peaked at 131.9 MPa when 3% steel fibers were incorporated. Compared to the P0G0 at 28 d, the compressive strengths of the 1%, 2%, and 3% steel fiber blended groups increased by 67%, 78.5%, and 93.4%, respectively. When comparing the 28 d strength to that at 3 d, the compressive strengths of the P0G1, P0G2, and P0G3 increased by 1.58, 1.42, and 1.17 times, respectively. The strength of CSUHPC can be further improved by blending PE fibers in addition to 1% steel fibers. At 28 d, compared to the 1% steel fibers alone, the compressive strengths of the P0.5G1, P1G1, and P1.5G1 increased by 3.7%, 10.6%, and 12.4%, respectively. The compressive strengths of the 0.5% and 1% PE fiber groups at 28 d increased by 3% and 4.8%, respectively, compared to the 2% steel fiber group alone.

**Figure 8:**
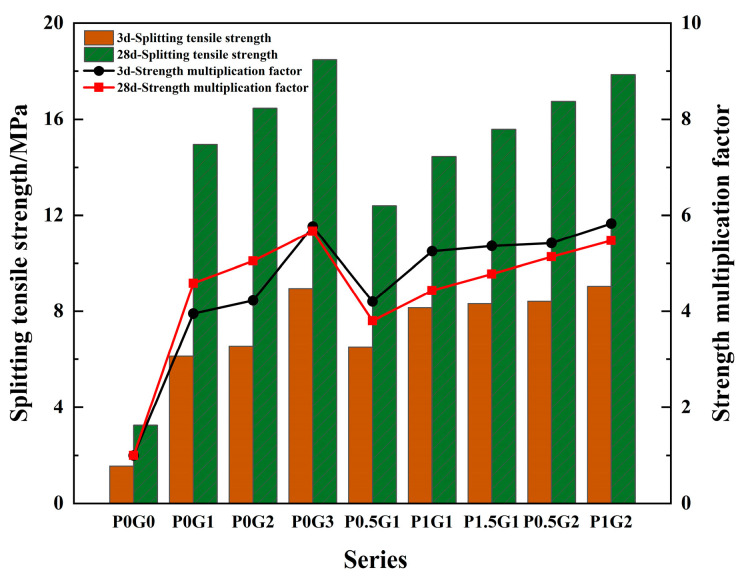
Splitting tensile strength.

As can be seen from Figure 8, the cube splitting tensile strength peaks at 18.5 MPa when 3% steel fibers are incorporated. Compared to the P0G0 group, the splitting tensile strength of the P0G1, P0G2, and P0G3 at 28 d increased by 3.59, 4.05, and 4.67 times, respectively. Compared to the 3 d, the strength of the P0G1, P0G2, and P0G3 at 28 d increased by 1.44, 1.51, and 1.07 times, respectively. Blending PE fibers with 1% steel fibers can further enhance the strength of cubic specimens. At 28 d, compared to the P0G1, blending 1.5% PE fiber groups increased the strength by 4.2%. At 28 d, compared to the P0G2, the P0.5G2 and P1G2 increased the strength by 1.7% and 8.5%, respectively.

At 3 d, the internal hydration reaction of CSUHPC specimens was not sufficient, and the matrix strength was in the rapid growth phase. However, at 28 d, the hydration reaction of the matrix was more complete, and the matrix strength began to stabilize. For the same content, the role of hybrid fibers in the compressive strength enhancement of CSUHPC was weaker than that of steel fibers alone, mainly owing to the high density of steel fibers, which disperse more easily in the cement paste. In contrast, the low density of PE fibers causes them to group together and float more readily. Additionally, during the specimen damage process, steel fibers can prevent crack propagation due to their high stiffness, while PE fibers, with their higher flexibility, contribute more to cracking. Moreover, the bond between steel fibers and the CSUHPC matrix is stronger than that of PE fibers. Additionally, coral sand absorbs a significant amount of unbound water during the CSUHPC mixing process. During the curing stage, the adsorbed free water is gradually released by the coral sand, which promotes sustained hydration reactions in UHPC at later ages. This mechanism allows the coral sand to exert an internal curing effect, thereby enhancing the long-term strength of CSUHPC.

#### 3.1.2. Tension–Compression Ratio and Strength Fitting

**Figure 9:**
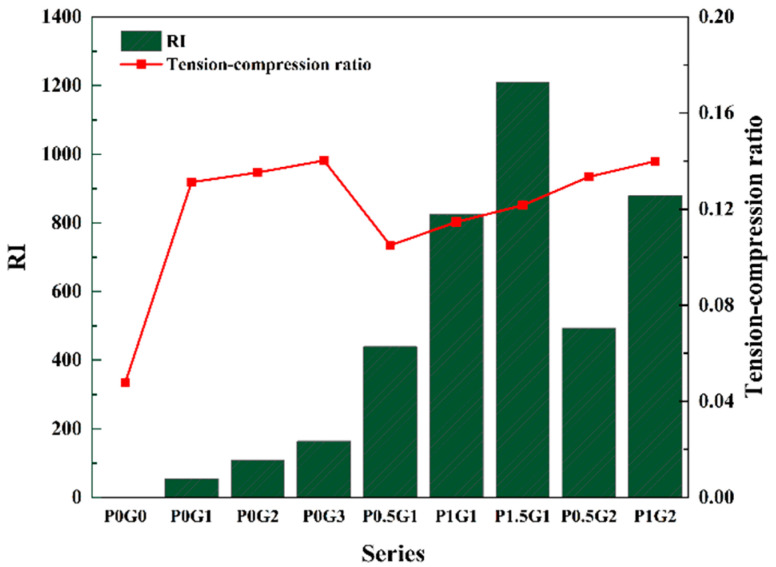
Tension–compression ratio.

**Figure 10:**
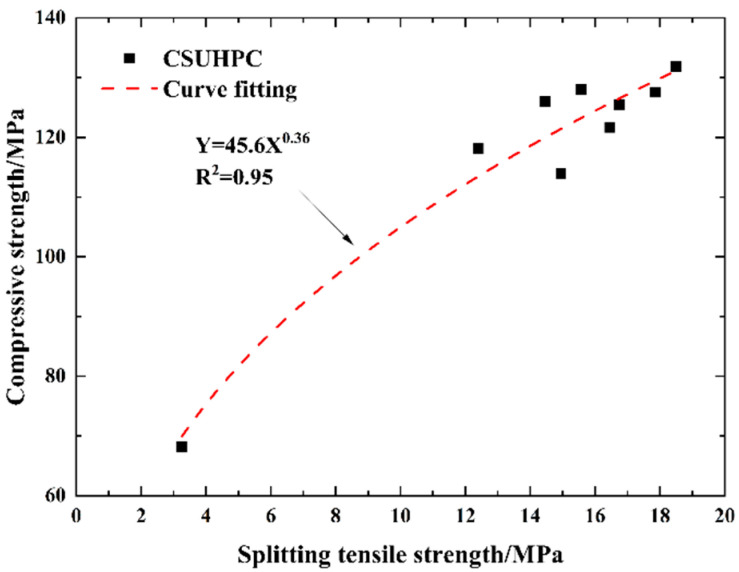
Strength fitting.

The values of the RI and the tension–compression ratio for different groups are shown in Figure 9. The PE fiber-reinforced group is discussed separately due to the small diameter of PE fibers, which results in large, calculated RI values. When the RI is 164, the CSUHPC specimen exhibits the largest tension–compression ratio, which is 1.9 times higher than that of P0G0. For specimens with single-doped steel fibers, the tension–compression ratio of CSUHPC increases with the rise in the RI. For mixed fibers, adding PE fibers to steel fibers further enhances the specimen toughness. Adding 1% PE fibers to 2% steel fibers increased the ratio by 3.5%. The relationship between the splitting tensile strength and compressive strength of CSUHPC is shown in Figure 10, and exhibits a good fit.

### 3.2. Stress–Strain Relationship of Cylindrical Specimens

#### 3.2.1. Failure Modes of Cylindrical CSUHPC Specimens

**Figure 11:**
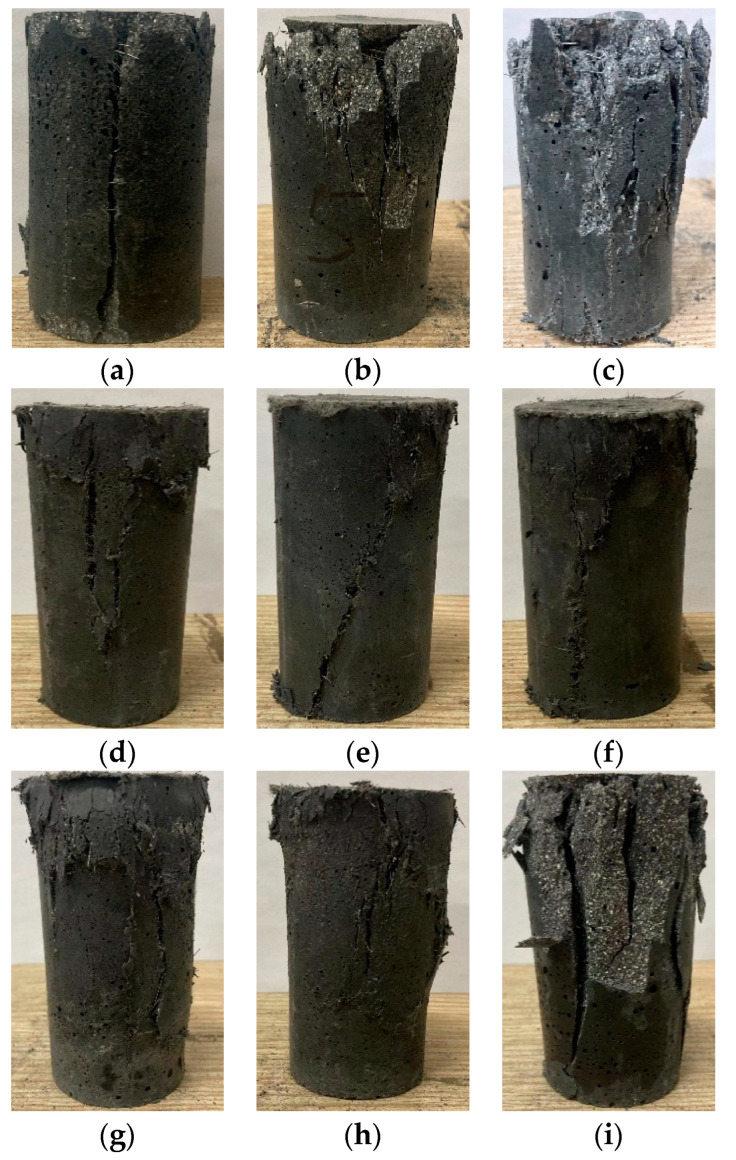
Failure modes of CSUHPC specimens under uniaxial compression with different fiber volume fractions: (**a**) P0G1; (**b**) P0G2; (**c**) P0G3; (**d**) P0.5G1; (**e**) P1G1; (**f**) P1.5G1; (**g**) P0.5G2; (**h**) P1G2; (**i**) P0G0.

The CSUHPC specimens in each group exhibited similar uniaxial damage morphology, as shown in Figure 11. During the initial stage of the test, the CSUHPC specimens were in the elastic stage, and no visible changes were discovered in the visible layer. As the test progressed and the specimen reached the limit of the linear elastic stage, microcracks started to appear in the apparent layer of the CSUHPC, with the crack propagation direction aligning with the direction of the applied load. As the load continued, the test block’s bearing capacity reached its limit, causing transverse lateral expansion in the middle of the specimen. However, the specimen did not undergo sudden failure due to the fiber reinforcement. Under continued loading, microcracks formed in the surface layer, which eventually led to the formation of macrocracks. Once the macrocracks appeared, the matrix’s bearing capacity weakened, the peak stress began to decrease, and the specimen eventually failed.

As shown in Figure 11, in the fiber-free specimen (P0G0) under compressive loading, some concrete blocks were dislodged, and macrocracks developed inside, as seen in Figure 11i. In the steel fiber-reinforced CSUHPC specimen, vertical macrocracks formed in the surface layer, some concrete blocks were dislodged, and the steel fibers bridged the cracks to control their width, as shown in Figure 11a–c. In the hybrid-fiber CSUHPC specimens, vertical macrocracks still formed in the surface layer, but the specimen maintained partial structural integrity owing to the bridging effect of the PE fibers.

#### 3.2.2. Complete Stress–Strain Curve

**Figure 12:**
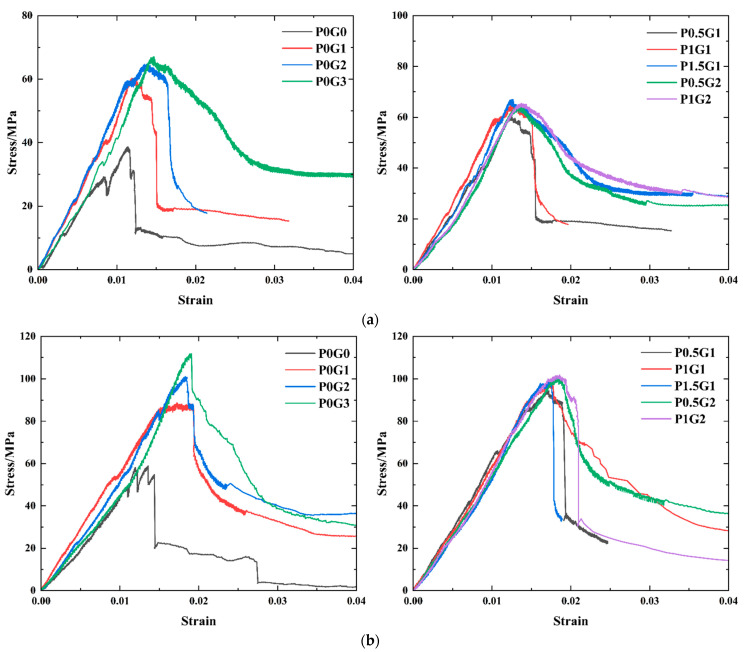
Stress–strain curves: (**a**) 3 d Curing age; (**b**) 28 d Curing age.

The 3 d and 28 d stress–strain curves of the CSUHPC cylindrical specimens (Φ50 mm × 100 mm) with different fiber contents are shown in Figure 12. From the figure, it can be observed that the compressive damage can be seen as three distinct phases: the elastic phase, the elastoplastic phase, and the plastic damage phase. In the early stage of loading, the stress–strain curve of the CSUHPC specimen exhibits linear elastic characteristics, and the incorporation of fibers effectively inhibits crack extension, thereby prolonging the elastic phase of the curve. When the elastic limit is reached, the stress–strain curve begins to grow nonlinearly, and it enters the descending phase after the stress reaches its peak value. The P0G0 exhibited brittle damage characteristics, as the curve started to drop steeply after the peak stress point. After fiber inclusion, the stress–strain curve still exhibits a sudden drop, but the fibers provide tensile strength, which imparts a certain toughness to the CSUHPC. Based on the P0G2 and P1G2 curves, under the same fiber content, the effect of single-type steel fibers on the specimen was weaker than that of the blended fibers. This indicates that hybrid fibers are more effective than single-type steel fibers in enhancing the toughness of CSUHPC.

At 3 d, the elastic phase curve is nonlinear, and the slope is lower than that at 28 d. This is mainly due to the hydration reaction of part of the cement in CSUHPC at this stage, as the matrix strength is undergoing rapid growth. In contrast, at 28 d, the curve of the elastic phase is linear, and the degree of cement hydration is higher, leading to a more stabilized matrix strength. From the stress–strain curve, the fiber incorporation effectively enhances the mechanical properties of CSUHPC specimens. The slope of the curve increases with the increase in fiber content before reaching the peak point.

#### 3.2.3. Effect of Fibers on the Mechanical Properties of Cylindrical CSUHPC Specimens

(1) Cylindrical specimen compressive strength and peak strain

**Figure 13:**
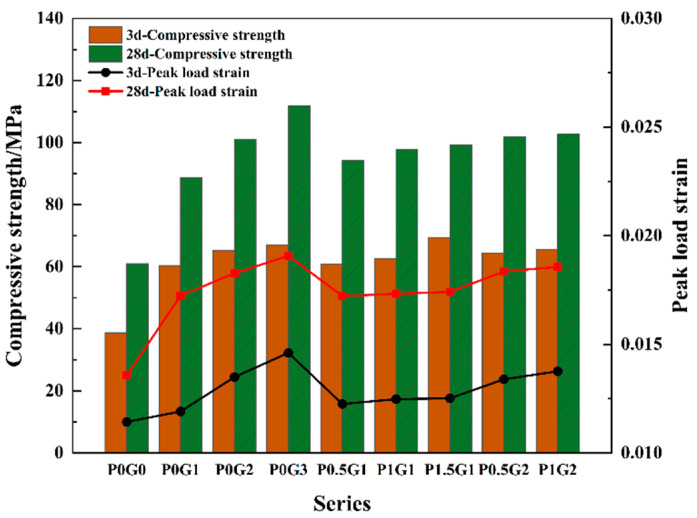
Compressive strength and peak load strain of cylindrical specimens.

The compressive strength versus peak strain for each group of CSUHPC is presented in Figure 13. As seen in the figure, the compressive strength of CSUHPC specimens with fiber addition is significantly higher than that of the P0G0. The strength increased with the content of steel fibers, and the strength of the CSUHPC was further enhanced by hybridizing PE fibers along with the steel fibers. During the damage process of the specimen, the fibers act as a bridge and inhibit crack propagation, thereby improving the strength of CSUHPC specimens. The cylindrical specimen with 3% steel fibers achieved a 28 d compressive strength of 111.9 MPa, although it was still lower than the compressive strength of the 100 mm cubes. At 28 d, the strength of the steel fiber group with 1%, 2%, and 3% steel fibers increased by 45.6%, 65.6%, and 83.4%, respectively, compared to the P0G0. The strength of P0G1, P0G2, and P0G3 increased by 47%, 54.9%, and 67%, respectively, compared to their 3d age. When PE fibers were hybridized with 1% steel fiber, the strength of CSUHPC increased with the content of PE fibers. At 28 d, when the PE fiber content reached 1.5%, the compressive strength showed an increase of up to 11.7%. In the case of 2% steel fiber mixed with 1% PE fiber, the compressive strength of CSUHPC increased by 1.7%. By comparing P0G2 with P1G1 and P0G3 with P1G2, it can be observed that steel fibers alone have a stronger effect on the compressive strength enhancement of CSUHPC specimens than hybrid fiber systems with the same fiber content.

As shown in Figure 13, it can be observed that the peak strain of each fiber-reinforced CSUHPC group is around 0.017, while the peak strain of the P0G0 is 0.013. This indicates that adding fibers into CSUHPC specimens improves their deformation resistance. The peak strains of the fiber-blended CSUHPC specimens showed a smaller increase compared to the specimens blended with steel fibers alone. At 28 d, the peak strains of the 1%, 2%, and 3% steel fiber-reinforced groups increased by 27%, 34.6%, and 40.6%, respectively, compared to the P0G0. Meanwhile, the peak strains of the steel fiber group specimens increased by 44.8%, 35.4%, and 30.6%, respectively, compared to the 3 d specimens. Under vertical loading, steel fibers resist axial deformation through their inherent stiffness. In contrast, PE fibers, despite their flexibility, mitigate lateral expansion by bridging microcracks in the CSUHPC matrix. Although vertical loading induces outward expansion in the specimen’s mid-region, PE fibers restrain crack propagation, preserving structural integrity. Therefore, steel fibers primarily resist axial deformation, whereas PE fibers are more effective at resisting lateral deformation [46].

(2) Compressive toughness and energy dissipation coefficient

**Figure 14:**
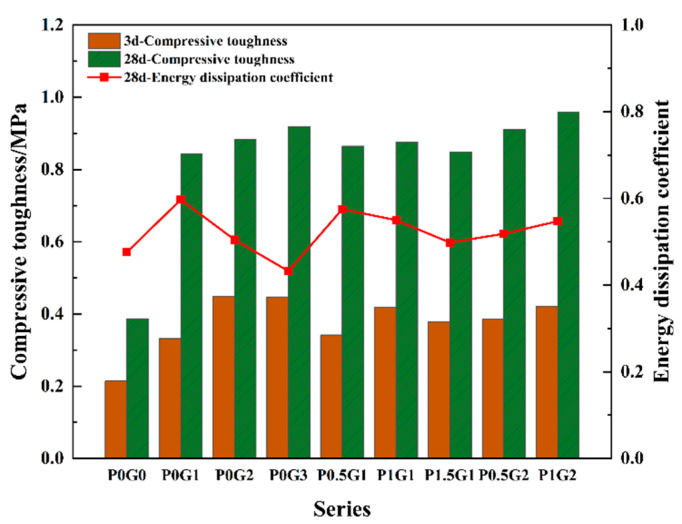
Compressive toughness and energy dissipation coefficient of cylindrical specimens.

From Figure 14, the compressive toughness of the CSUHPC increases with the addition of fiber blending and the growth of the curing age. The toughness of the CSUHPC reached its peak value by mixing 1% PE fibers with 2% steel fibers, which led to a 1.48-fold increase compared to the P0G0. At 28 d, compared to the P0G0, the toughness of CSUHPC with 1%, 2%, and 3% fiber contents increased by 1.18, 1.28, and 1.38 times, respectively. At 28 d, the compressive toughness of specimens with 1%, 2%, and 3% steel fibers increased by 1.54, 0.97, and 1.06 times, respectively, compared to the 3d. Blending 0.5%, 1%, and 1.5% PE fibers with 1% steel fiber resulted in a compressive toughness increase of 2.5%, 3.9%, and 0.5%, respectively. Blending 0.5% and 1% PE fibers with 2% steel fibers enhanced the toughness by 3% and 8.6% compared to the P0G2, respectively. Fiber blending significantly increased the strength and peak strain of CSUHPC, while the slope of the pre-peak section also varied with fiber combinations.

The energy dissipation coefficient refers to the energy dissipated by concrete during the application of force. As shown in Figure 14, the maximum energy dissipation occurs when 1% steel fibers are added, and the energy dissipation coefficient increases by 25.4% compared to the P0G0. By observing the line graph, as the steel fibers content increases, the energy dissipation coefficient generally decreases. When 1% steel fibers are blended with PE fibers, the energy dissipation coefficient decreases with the increase in PE fiber content. In contrast, when 2% steel fibers are combined with PE fibers, the energy dissipation coefficient shows an increasing trend. By examining the stress–strain curve, as the steel fiber content increases, the post-peak region of the curve results in a decline in the energy dissipation coefficient. In the case of 1% steel fiber blended with PE fibers, the slope of the curve before the peak stress does not change significantly, but the descending section of the curve becomes more gradually softening, leading to a reduction in the energy dissipation coefficient. In contrast, with 2% steel fiber blended with PE fibers, the descending section of the curve shows a rapid strength degradation, which results in an enhancement in the energy dissipation coefficient.

### 3.3. Compressive Constitutive Relationship of CSUHPC Specimens

**Figure 15:**
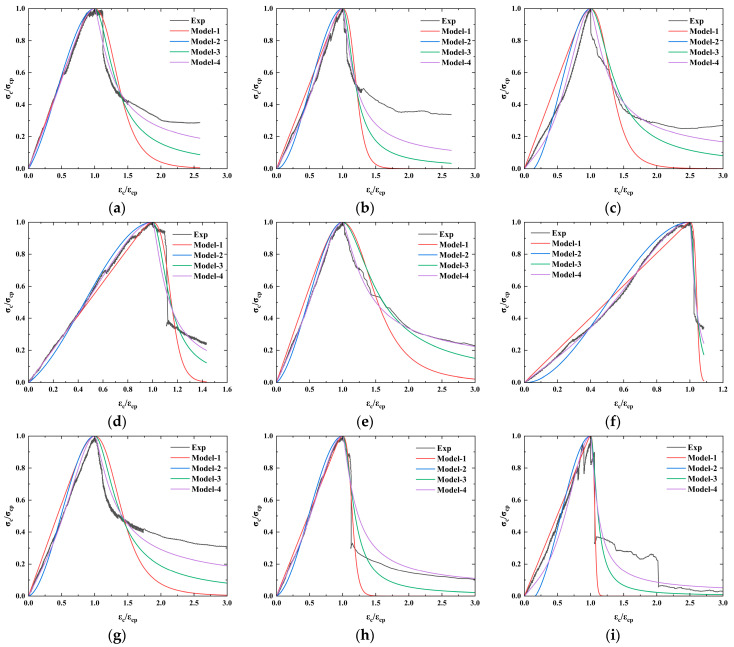
Comparison of constitutive model fitting curves and experimental curves: (**a**) P0G1; (**b**) P0G2; (**c**) P0G3; (**d**) P0.5G1; (**e**) P1G1; (**f**) P1.5G1; (**g**) P0.5G2; (**h**) P1G2; (**i**) P0G0.

Table 4 below lists the fitting parameters of the constitutive models. The comparison between the predicted curve and the test curve is presented in Figure 15.

By comparing the experimental curve with the simulation curves, it is found that the unified constitutive model exhibits lower fitting accuracy compared to the piecewise constitutive models. Among the segmented constitutive models, Model-2, Model-3, and Model-4 demonstrate satisfactory fitting performance for the ascending branch of the curve, with Model-2 being relatively less accurate. During the descending branch fitting process, Model-4 achieves the best agreement. In conclusion, the Deju Zhu model provides the optimal simulation.

### 3.4. Comprehensive Performance Assessment of CSUHPC Specimens

**Figure 16:**
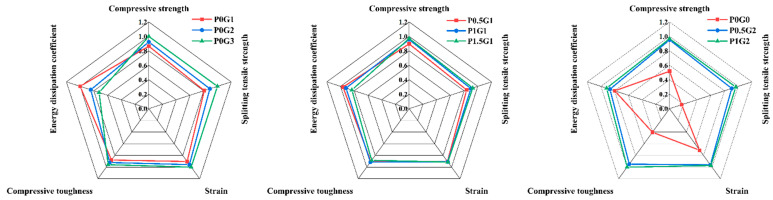
Radar chart.

The radar chart in Figure 16 evaluates the strength of the CSUHPC.

The performance evaluation of CSUHPC specimens with different fiber contents is summarized in Table 5. The group with the best overall performance is P1G2, followed by P0G3.

## 4. Conclusions

This study aims to address the low strength and poor durability issues of structures in tropical island environments. UHPC was prepared using coral aggregate. The changes in capabilities of the CSUHPC were investigated by adjusting the fiber type and content, and the optimum content of fibers was determined in conjunction with radar charts.

(1) Experimental results from cubic compressive and splitting tensile tests demonstrated that fiber significantly enhances the mechanical strength of CSUHPC. Specifically, at a steel fiber content of 3%, the CSUHPC specimens achieved peak compressive strength and splitting tensile strength of 131.9 MPa and 18.5 MPa, respectively. Furthermore, the 3% steel fiber-reinforced CSUHPC exhibits the highest toughness, with its tension–compression ratio showing a 1.9-fold increase compared to the P0G0.
(2) Compressive stress–strain tests demonstrated that fiber incorporation induces a transition in CSUHPC specimen failure modes from brittle to ductile. At a 3% steel fiber volume fraction, CSUHPC achieved a peak stress of 111.9 MPa and a peak strain of 0.019, showing increases of 83.4% and 40.6%, respectively, compared to P0G0. The hybrid fiber system of 2% steel fibers and 1% PE fibers exhibits optimal compressive toughness, exhibiting a 1.48-fold enhancement relative to P0G0. Additionally, CSUHPC specimens with 100 mm cubes exhibit higher compressive strength than Φ50 mm × 100 mm cylindrical specimens.
(3) Comparative analysis of compressive constitutive equations revealed that piecewise models demonstrated superior fitting accuracy for CSUHPC over unified constitutive models, with the Deju Zhu model exhibiting the optimal performance.
(4) A multivariate evaluation system was established, and radar charts were used for the comprehensive evaluation of CSUHPC specimens. The analysis concluded that the comprehensive performance of the CSUHPC specimens was optimal when the PE fiber content was 1% and the steel fiber content was 2%.

This study has several limitations that merit further investigation in future research within the region:

(1) The tropical island and reef regions are characterized by a combined extreme environment of high temperature, high salinity, intense radiation, and high humidity. Future research must simulate prioritizing these realistic service conditions to explore the performance of CSUHPC under varied exposure scenarios.
(2) Additionally, given the service life of structures in these regions is significantly shortened due to environmental degradation, durability enhancement strategies for CSUHPC must be prioritized in subsequent studies.

## Figures and Tables

**Table 1:** Chemical composition table of coral sand/%.

Ingredient	CaO	SiO_2_	Al_2_O_3_	Fe_2_O_3_	SrO	MgO	Na_2_O	SO_3_	K_2_O	TiO_2_
Content	85.25	6.06	2.43	2.07	1.91	0.50	0.46	0.43	0.35	0.29

**Table 2:** Different fiber physical properties.

Fiber Type	Diameter/mm	Length/mm	Tensile Strength/MPa	Density/g·cm^−3^
Steel Fiber	0.22	12	3000	7.85
PE Fiber	0.0156	12	3980	0.97

**Table 3:** Mix proportions.

Series	Mass Ratio							PE Fiber (V_*f*_)	Steel Fiber (V_*f*_)
	Water	Cement	Silica Fume	Glass Microspheres	Coral Sand	Water Reducer	Defoamer		
P0G0	0.23	0.7	0.15	0.15	1	0.015	0.0015	0	0
P0G1	0.23	0.7	0.15	0.15	1	0.015	0.0015	0	1
P0G2	0.23	0.7	0.15	0.15	1	0.015	0.0015	0	2
P0G3	0.23	0.7	0.15	0.15	1	0.015	0.0015	0	3
P0.5G1	0.23	0.7	0.15	0.15	1	0.015	0.0015	0.5	1
P1G1	0.23	0.7	0.15	0.15	1	0.015	0.0015	1	1
P1.5G1	0.23	0.7	0.15	0.15	1	0.015	0.0015	1.5	1
P0.5G2	0.23	0.7	0.15	0.15	1	0.015	0.0015	0.5	2
P1G2	0.23	0.7	0.15	0.15	1	0.015	0.0015	1	2

The mix proportion in this study adopts a mass ratio system, with the mass of coral sand set as 1. The remaining components in CSUHPC are proportioned by their mass relative to that of coral sand. Fibers are incorporated using the volume-based addition method, maintaining a constant volume fraction per unit volume of concrete. P0G0 (0% PE fiber + 0% steel fiber).

**Table 4:** Constitutive model fitting parameters.

Series	Model-1	Model-2		Model-3		Model-4	
	*n*	*a*	*b*	*a*	*b*	*a*	*b*
P0G0	69.18	−0.63	84.62	0.48	84.62	0.48	21.06
P0G1	8.69	0.65	10.72	1.07	10.72	1.07	5.77
P0G2	16.76	−0.03	28.35	0.76	28.35	0.76	10.23
P0G3	9.95	−0.53	8.52	0.50	8.52	0.50	5.64
P0.5G1	25.03	0.55	55.07	1.03	55.07	1.03	18.55
P1G1	6.14	0.30	4.26	0.92	4.26	0.92	3.95
P1.5G1	116.26	−0.03	734.42	0.77	734.42	0.77	107.96
P0.5G2	7.59	0.12	8.57	0.82	8.57	0.82	4.87
P1G2	25.56	0.19	33.13	0.88	33.13	0.88	9.22

**Table 5:** Multi-criteria performance assessment of CSUHPC using radar chart visualization.

Series	*S_i_*	*C_i_*	*A_i_*	*B_i_*	*F_i_*
P0G0	0.5883	3.4502	0.2660	0.6207	0.4064
P0G1	1.8865	5.2516	0.8530	0.8591	0.8561
P0G2	1.9566	5.3393	0.8847	0.8621	0.8733
P0G3	2.0807	5.5466	0.9408	0.8495	0.8940
P0.5G1	1.9266	5.2959	0.8711	0.8628	0.8669
P1G1	2.0097	5.4055	0.9087	0.8639	0.8860
P1.5G1	1.9490	5.3301	0.8812	0.8616	0.8714
P0.5G2	2.0424	5.4554	0.9235	0.8620	0.8922
P1G2	2.2116	5.6728	1.0000	0.8632	0.9291

## Data Availability

Date are contained within the article.
